# The mediating effect of sleep quality and fatigue between depression and renal function in nondialysis chronic kidney disease: a cross-sectional study

**DOI:** 10.1186/s12882-022-02757-z

**Published:** 2022-03-31

**Authors:** Ya-Fang Ho, Pei-Ti Hsu, Kai-Ling Yang

**Affiliations:** 1 School of Nursing, China Medical University, No. 100, Sec. 1, Jingmao Rd., Beitun Dist, Taichung City, 406040 Taiwan ROC; 2 Department of Nursing, Ching Kuo Institute of Management and Health, Keelung, Taiwan, ROC; 3 Nephrology Medicine, China Medical University Hospital, Taichung, Taiwan, ROC

**Keywords:** Chronic kidney disease, Depression, Fatigue, Sleep quality, Renal function

## Abstract

**Background:**

Depressive symptoms, fatigue, and poor sleep quality are associated with renal function deterioration in patients with nondialysis chronic kidney disease (CKD-ND). This study was designed to examine whether fatigue and sleep quality are mediators of the association between depression and renal function.

**Methods:**

This study adopted a cross-sectional study design. Patients with CKD-ND aged 20 years or older were recruited by purposive sampling at a medical center in Central Taiwan from December 2020 to July 2021. Data were collected using the Emotional and Social Support Scale, Fatigue Scale, Beck Depression Inventory-II (BDI-II), and Pittsburgh Sleep Quality Index. Medical records were reviewed to obtain the estimated glomerular filtration rate (eGFR) for the next month. The relationships among variables were analyzed using structural equation modeling to assess the goodness-of-fit of the model. Then, the bootstrapping method was used to analyze the mediated effect.

**Results:**

Two hundred forty-two participants (mean age 70.5 years and 53% males) were included in the analysis. About 39% of the participants met the criteria for depressive symptoms in BDI-II, and 91% reported having sleep disturbances. Participants’ degree of fatigue was not high (20.4 ± 13.3). The average eGFR was 25.45 mL/min/1.73 m ^2^ (± 13.36). The results showed that fatigue, sleep quality, and eGFR were significantly correlated with depression. The total effect size was − 0.8304 (95% confidence interval [CI], − 0.9602 to − 0.7006), and the indirect effect size was − 0.1738 (95% CI, − 0.2812 to − 0.0651), which was a statistically significant difference, indicating that the model has a mediating effect. According to mediation analysis, fatigue and sleep quality had a significant indirect effect on the relationship between depression and renal function (95% CI, − 0.0587 to − 0.0039).

**Conclusions:**

The findings suggest that fatigue and poor sleep quality may mediate the association between depression and renal function.

## Background

Chronic kidney disease (CKD) is one of the reasons for the increasing global burden of noncommunicable diseases [1]. CKD has a global prevalence of approximately 10%–13% [2]. It indirectly influences the mortality of the global population by increasing the risk of cardiovascular diseases [3] and further affects patients’ quality of life [4–6]. When CKD progresses to end-stage renal disease (ESRD), patients must undergo renal replacement therapy to maintain life. Therefore, how to delay the progression of nondialysis chronic kidney disease (CKD-ND) is an important issue that needs to be addressed.

Although traditional risk factors such as cardiovascular disease, proteinuria, and hyperglycemia promote renal function deterioration in patients with CKD-ND [7–9], recent studies have focused on psychological factors such as depression [10]. Depression is common in patients with CKD-ND and considerably affects the disease outcome [6, 10]. Studies have shown that CKD-ND patients with depression have a significantly higher risk of undergoing dialysis or death than those without depression [11, 12]. Poor sleep quality is also a common problem of patients with CKD-ND [9, 13, 14]. Short and low-quality sleep is a risk factor for the worsening of CKD, and more interruptions in sleep and shorter sleep duration are related to a sharp drop in estimated glomerular filtration rate (eGFR) and an increase in proteinuria [5, 15]. Sleep disorders and depression are closely related and complex and may influence each other [16]. Studies have highlighted that sleep problems usually precede depression [17], and poor sleep quality is an independent risk factor for depression [18]. Some studies indicate that depression is a significant predictor of poor sleep quality [14, 19]. Sleep quality and depression have been confirmed to be correlated; however, their causal relationship remains controversial, and it is even possible that both coexist [20]. Fatigue is among the most common symptoms experienced by patients with CKD-ND [21–23]. CKD-ND-related fatigue has a complex multifactorial etiology [22, 23], including low albumin levels, anemia, and restless legs syndrome [21, 23]. Some studies also found that fatigue may be related to obstructive sleep apnea (OSA) [23, 24]. Many psychosocial factors are related to the fatigue experienced by patients with CKD-ND, including depression, subjective sleep quality, and anxiety [21–23]. Moreover, fatigue is independently associated with ESRD progression in patients with CKD-ND [22, 23]. Nocturnal hypoxia caused by OSA also affects renal function [7, 24].

A study has reported that the continuous increase in depression scores over time was associated with an increased risk of death and cardiovascular disease. However, the strength of this relationship weakened after including the time interval in the statistical analysis [25]. Therefore, it is reasonable to infer that the relationship between depression and poor prognosis should be influenced by mediating factors rather than depression directly leading to poor prognosis. So far, studies have explored the relationship between depression, sleep quality, and eGFR [5, 11, 12, 15]. Theoretically, the association between depression and sleep disturbances is instead a complex bidirectional one [25]. Nonetheless, related studies have found that impaired sleep continuity and disinhibition of rapid eye movement (REM) sleep in patients with depression lead to sleep problems [25, 26]. Schønning et al. performed short-term psychoanalytic psychotherapy on adolescents with depressive symptoms and found that sleep disturbances improved further after treatment for depression [27]. Fatigue and depression are complex constructs, they have been strongly correlated [28]. Although fatigue is common in the CKD-ND population, few studies have explored the relationship between fatigue and renal function. Considering the above findings, we hypothesized that fatigue and sleep quality may mediate the relationship between depression and renal function in patients with CKD-ND. Therefore, this study aimed to examine whether fatigue and sleep quality are mediators of the association between depression and renal function among patients with CKD-ND.

## Methods

### Design and participants

**Fig. 1:**
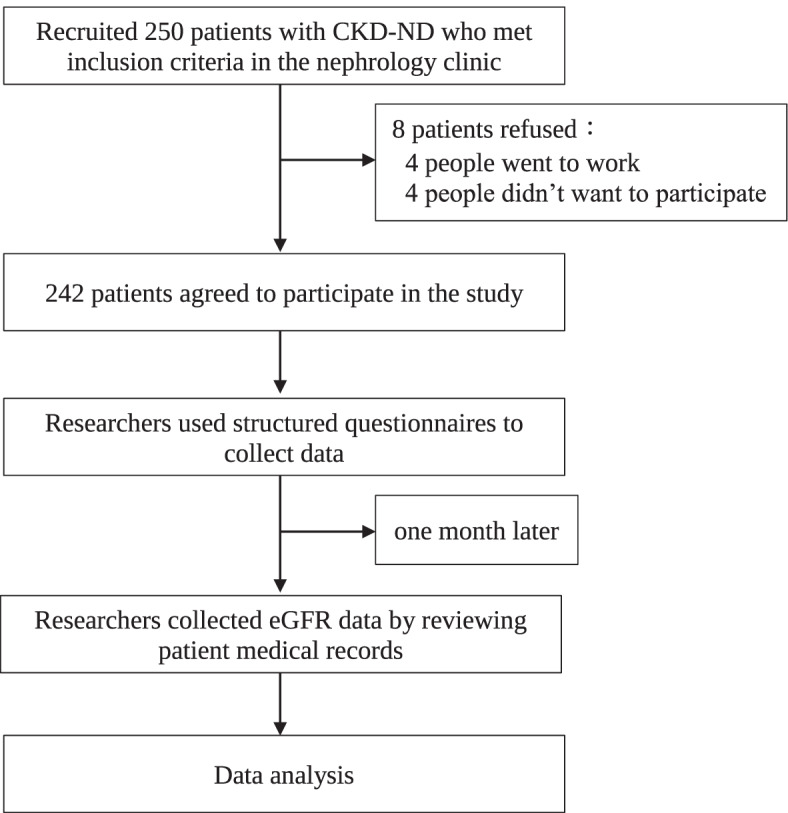
The research process

A cross-sectional survey was conducted. On the basis of the inclusion criteria, a convenience sample of 242 eligible patients with CKD-ND was recruited from a nephrology clinic at a medical center in Central Taiwan from December 2020 to July 2021. The participants were provided information about the study. The inclusion criteria were as follows: (1) patients aged 20 years or above, (2) those diagnosed with CKD with an eGFR of ≤ 45 mL/min/1.73 m^2^, and (3) those with intact cognition who could communicate in Mandarin or Taiwanese. The exclusion criteria were as follows: (1) patients with ESRD undergoing dialysis; (2) those with dementia, severe mental illness, or intellectual disabilities; (3) those who have severe complications, such as arrhythmia or advanced heart failure; and (4) those who have cancer. After written informed consent was collected, the researcher conducted face-to-face interviews using a structured questionnaire, and patients’ renal function data were collected by reviewing their medical records. The research process is shown in Fig. 1. We followed the consolidated checklist for reporting cross-sectional studies (STROBE).

The sample size was calculated using G*Power 3.1. In linear multiple regression, a fixed model and R2 deviation from zero were selected as the statistical method, and the effect size was set to 0.15 (medium effect size), with an α value of 0.05 and power of 0.8. The estimated sample size was 160. However, considering that the sample loss rate was less than 20%, it was estimated that 210 patients will be recruited. A total of 250 eligible patients with CKD-ND were contacted. Eight eligible participants declined to participate because of time constraints and unwillingness. There were no missing data during the analysis.

### Measures

Several questionnaires were used to measure the research variables, including demographics, social support, depression, fatigue, and sleep quality. The medical records were also reviewed to obtain the eGFR for the next month. According to a previous study, social support is a factor affecting patients’ depression state [29]. Thus, it was treated as a potentially confounding variable in this study. The corresponding questionnaires included personal characteristics, Emotional and Social Support Scale (ESS), Beck Depression Inventory-II (BDI-II), Fatigue Scale (FS), Pittsburgh Sleep Quality Index (PSQI), and eGFR. A detailed description of each questionnaire is provided below.

### Personal characteristics

The information on patients’ personal characteristics was self-reported, including age, sex, marital status, educational level, occupation, and living conditions. The CKD stage was collected by reviewing patients’ medical records.

### Social support

Lu’s Chinese version of the ESS was used as the social support scale in this study. It was designed to assess the degree of self-perceived levels of actual emotional and social support. The ESS comprises six items scored using a 4-point Likert scale with minimum and maximum total scores of 6 and 24, respectively. The higher the score on the scale, the better the emotional and social support of the individual. The Cronbach’s α value for the social support scale in one reference study was 0.72, and the content validity index was 0.86 [30]. The Cronbach’s α value of the scale in this study was 0.94, indicating that the questionnaire results are highly reliable.

### Fatigue

FS was developed by Lin et al. and adopts the self-reporting method [31]. This scale assesses the fatigue state of patients undergoing hemodialysis and comprises five subscales with 26 items: decreased vigor and motivation (eight items), decreased physical ability (five items), decreased mental ability (five items), decreased daily activities (four items), and feeling down and lost control (four items). The participants were instructed to select the frequency of occurrence of the feeling or behavior that they had for the past month. The items were scored using a 4-point Likert scale (0 = rarely or never occurred, 1 = occurred sometimes, 2 = occurred frequently, and 3 = occurred almost every day), and the total score ranged between 0 and 78 points. The higher the score, the more severe the fatigue state. The Cronbach’s α value for the entire scale was 0.91, and those of the five subscales ranged between 0.72 and 0.85; regarding validity, the FS has a significant positive correlation with the Visual Analog Fatigue Scale (*r* = 0.64; *p* < 0.01) [31]. The Cronbach’s α value in this study was 0.94, and those of the five subscales ranged between 0.72 and 0.90.

### Depression

The BDI is a well-established sensitive screening tool for measuring the severity of depressive symptoms in the past 2 weeks. It is a 21-item self-report questionnaire, and each item is scored using a 4-point Likert scale. The value assigned to each answer ranges from 0 to 3 (0 = not at all to 3 = severely), and the total score ranges between 0 and 63 points. The higher the score, the higher the severity of depression. The test–retest reliability and Cronbach’s α value of BDI were 0.93 and 0.91, respectively [32]. The Chinese version of BDI-II was examined by Lu et al. [32]. The Cronbach’s α value of the scale was 0.94; regarding validity, BDI-II has a significant positive correlation with the Chinese Health Questionnaire (*r* = 0.69; *p* < 0.001) [33]. The Cronbach’s α value in this study was 0.92. In the Chinese version of BDI-II, total scores of 0–16, 17–22, 23–30, and 31–63 indicate the normal range, mild depression, moderate depression, and severe depression, respectively [33].

### Sleep quality

The PSQI is a self-reported questionnaire that assesses an individual’s subjective sleep quality over a 1-month period. It consists of 19 items divided into seven sections, including subjective sleep quality, sleep latency, sleep duration, habitual sleep efficiency, sleep disturbances, use of sleeping medications, and daytime dysfunction. The score for sleep quality ranges from 0 to 21, with higher scores indicating poorer sleep quality. A global score of more than 5 represents poorer sleep quality, and a score of ≤ 5 is considered good sleep quality. The internal consistency reliability of the scale is 0.83 [34]. Tsai et al. conducted a Chinese version of the PSQI test on 87 participants with primary insomnia and 157 healthy controls, and the scale reliability was 0.82–0.83, and the test–retest reliability after 2 weeks ranges from 0.77 to 0.85; moreover, primary insomnia and healthy groups have significant discriminant differences (*p* < 0.001) [35]. The Cronbach’s α value of the scale in this study was 0.82.

### Renal function estimation

Renal function was assessed using eGFR. After collecting the structured questionnaires, the medical records were reviewed to obtain the eGFR for the next month. The eGFR is the most accurate measure of renal function [36, 37]. This study used the simplified Modification of Diet in Renal Disease (MDRD) to estimate the eGFR. It is currently the most popular method to evaluate renal function in clinical practice [36]. Although MDRD easily underestimates eGFR in healthy individuals [38], our study participants were patients with CKD-ND. Furthermore, the CKD Epidemiology Collaboration (CKD-EPI) estimated eGFR closest to the directly measured GFR in patients with eGFR of ≥ 90 mL/min/1.73 m^2^, whereas MDRD had excellent accuracy when the eGFR ranges from 15 mL/min/1.73 m2 to 29 mL/min/1.73 m^2^ (in our study, the average eGFR was 25 mL/min/1.73 m^2^) [39]. There are no differences in accuracy between MDRD and CKD-EPI [39, 40].

The eGFR was calculated separately for men and women using the MDRD: GFR (expressed in mL/min/1.73 m^2^) = 186 × [SCr (mg/dl)] − 1.154 × (age) − 0.203 × (0.742 if female) × 1.212 (if black), where SCr is serum creatinine (milligrams per deciliter).

### Ethical considerations

All procedures were approved by the Research Ethics Committee of a medical university hospital in Central Taiwan (CMUH109-REC1-128). The study purpose was explained to potential participants who met the inclusion criteria, and written informed consent was obtained from all participants.

### Statistical analysis

Statistical analyses were performed using the Statistical Package for the Social Sciences version 25.0 (IBM Corp. Armonk, NY, USA). Descriptive statistics were obtained for the variables used. One-way analysis of variance and independent t-test were conducted to examine whether there were significant differences in sociodemographic variables, depression, fatigue, and sleep quality between groups. Pearson’s correlation was used to analyze whether there were any associations between variables.

IBM SPSS AMOS 22.0 (IBM Corp. Armonk, NY, USA) was adopted to calculate the goodness-of-fit of the model using the goodness-of-fit index (GFI), average GFI (AGFI), and root mean square error of approximation (RMSEA). A model was considered a good fit if the GFI and AGFI values were ≥ 0.90 and the RMSEA value was ≤ 0.08. Then, the bootstrapping method was used to analyze the mediated effect.

## Results

### Distribution of personal characteristics and differences in depression, fatigue, and sleep quality

Two hundred forty-two patients were enrolled in this study. Table 1 shows the personal characteristics and ESS of all participants and the relationship between personal characteristics, depression, fatigue, and sleep quality of participants. The mean age of subjects was 70.5 ± 13.3 years; 47% were women and 53% were men. Regarding marital status, 77% of subjects were married, and 15% were widowed. Regarding educational level, 85 (35%) subjects had an education level below elementary school. About 81% were unemployed, 37% were living with spouse and children, and 29% were living with spouse only. More than 90% of subjects had religious beliefs. Regarding disease status, most patients (*n* = 106; 44%) had stage 3 CKD, and the number of patients with stage 4 and 5 CKD-ND was 67 (28%) and 69 (29%), respectively. The average eGFR was 25.45 mL/min/1.73 m^2^ (± 13.36). The average ESS score was 19.1 points (total score ranged from 6 to 24 points), which showed that the research subjects had good emotional and social support. The results of the analysis of the relationship between personal characteristics, depression, fatigue, and sleep quality showed statistically significant differences in marital status, religious beliefs, living conditions, CKD stage, and ESS scores.

**Table 1:** Differences in personal characteristics, depression, fatigue, and sleep quality (*n* = 242)

Variable	n (%)	BDI-II		FS		PSQI	
		Mean (SD)	t/F/r	Mean (SD)	t/F/r	Mean (SD)	t/F/r
**Age in years, M (SD)**	70.5(13.3)		-0.02		0.09		0.05
**Gender**			-1.00		-2.25		-1.53
Male	128(53%)	14.9(10.8)		19.6(13.3)		10.2(4.0)	
Female	114(47%)	16.3(10.2)		21.4(14.0)		11.0(3.8)	
**Education**			0.14		3.13		0.96
Elementary or below	85(35%)	15.1(10.3)		20.3(12.9)		10.3(3.7)	
Junior high school	44(18%)	16.0(9.7)		21.6(13.8)		11.0(4.2)	
High school	59(25%)	15.4(10.6)		19.1(12.3)		11.1(3.8)	
College or above	54(22%)	16.1(11.7)		19.9(12.8)		10.1(4.3)	
**Employment**			0.58		-0.60	10.6(4.0)	1.47
Unemployed	196(81%)	15.7(10.0)		20.3(13.3)		10.7(4.0)	
Employed	46(19%)	14.7(12.7)		22.2(12.8)		9.8(3.7)	
**Marital status**			2.48		6.36**		5.80**
Married/cohabitating	185(77%)	14.8(10.4)		18.9(12.4)		10.2(3.8)	
Single	20(8%)	16.5(13.0)		20.8(14.5)		9.9(4.1)	
Widowed/divorced	37(15%)	18.9(9.5)		27.3(14.7)		12.5(4.3)	
**Religious beliefs**			-3.64***		-4.17***		-2.18*
Yes	224(93%)	14.9(10.1)		14.5(9.9)		10.4(3.9)	
No	18(7%)	24.1(11.6)		21.8(13.6)		12.5(4.1)	
**Living condition**			2.74*		3.94**		3.52**
Alone	23(10%)	16.7(10.2)		21.3(13.3)		10.4(4.5)	
With spouse	71(29%)	16.5(10.9)		20.9(14.1)		10.2(3.9)	
With children	24(10%)	17.6(10.7)		24.2(16.1)		12.3(3.7)	
With spouse and children	90(37%)	12.6(9.5)		16.7(10.4)		9.9(3.7)	
With caregiver	20(8%)	19.7(8.8)		29.4(11.0)		13.2(3.7)	
With parents	14(6%)	18.4(14.0)		21.5(15.9)		10.3(4.1)	
**CKD stage**			158.05***		24.32***		48.17***
Stage 3	106(44%)	9.9(6.5)		15.8(12.5)		8.8(3.4)	
Stage 4	67(28%)	11.6(6.9)		19.1(11.9)		9.9(4.0)	
Stage 5	69(29%)	28.1(7.7)		28.8(11.7)		13.8(2.5)	
**eGFR, M (SD)**	25.45(13.4)		-0.64**		-0.39**		-0.50***
**ESS, M (SD)**	19.1(4.9)		-0.16**		-0.22**		-0.28**

^*^p < 0.05; **p < 0.01; ***p < 0.001; *CKD* Chronic kidney disease, *GFR* Glomerular filtration rate, *ESS* Emotional social support scale, *BDI-II* Beck Depression Inventory-II, *PSQI* Pittsburgh Sleep Quality Index, *FS* Fatigue scale, *t*: Independent t-test, *F* One-way analysis of variance, *r*: Pearson’s correlation

In Table 1, patients with stage 5 CKD-ND had clinically significant depression (defined as BDI-II ≥ 17), and patients with all stages of CKD had poor sleep quality (defined as PSQI > 5). Most especially, patients with stage 5 CKD-ND had a score of 13.8 (± 2.5). The degree of fatigue of patients with all stages of CKD-ND was not high (total score 0– 78), but patients with stage 5 disease still had a higher fatigue score than other stages.

### Distribution of CKD stage in depression, fatigue, and sleep quality

Table 2 shows that the average BDI-II score of the study participants was 15.6 ± 10.5, which indicated that the depression degree of study participants was in the normal range. Approximately 39% of participants met the criteria for depressive symptoms of BDI-II: 31 (13%) and 34 participants (14%) were categorized as having mild and moderate depressive symptoms, respectively. Twenty-eight participants (12%) were classified as having major depressive symptoms, all of whom had stage 5 CKD-ND. The average PSQI score was 10.55 ± 3.96. A total of 220 participants (91%) reached the standard for sleep disturbances. Patients with all stages of CKD-ND had poor sleep quality, especially those with stage 5 CKD-ND. In the subscale, sleep duration had the highest score (1.9 ± 0.71), followed by sleep latency (1.87 ± 0.87), habitual sleep efficiency (1.75 ± 1.10), and subjective sleep quality (1.61 ± 0.75). This finding indicated that the participants had short sleep duration, took a long time to fall asleep, had poor habitual sleep efficiency, and subjectively thought that their sleep quality was poor. The average FS score was 20.4 ± 13.3, indicating that participants’ degree of fatigue was not high. Among the five subscales, the decreased vigor and motivation subscale had the highest average score (6.95 ± 4.8).

**Table 2:** BDI-II, fatigue and PSQI score and distribution

Variable	Total(*n* = 242)		Stage 3 *n* = 106	Stage 4 *n* = 67	Stage 5 *n* = 69
	Mean (SD)	n (%)	n (%)	n (%)	n (%)
**BDI-II total**	15.6(10.5)				
Normal range (0–16)		149(61%)	91(86%)	53(79%)	5(7%)
Mild depression (17–22)		31(13%)	9(8%)	9(13%)	13(19%)
Moderate depression (23–30)		34(14%)	6(6%)	5(8%)	23(33%)
Severe depression (31–63)		28(12%)	0(0%)	0(0%)	28(41%)
**PSQI total**	10.55(3.96)				
Subjective sleep quality	1.61(0.75)				
Use of sleep medication	1.10(1.26)				
Sleep latency	1.87(0.87)				
Sleep duration	1.90(0.71)				
Habitual sleep efficiency	1.75(1.10)				
Subjective sleep disturbances	1.21(0.45)				
Daytime functioning	1.12(0.80)				
Good sleep quality (≦5)		22(9%)	17(16%)	5(7%)	0(0%)
Poorer sleep quality (> 5)		220(91%)	89(84%)	62(93%)	69(100%)
**Fatigue scale**	20.41(13.3)				
Vigor and motivation	6.95(4.8)				
Physical ability	2.99(3.3)				
Mental ability	4.61(2.8)				
Daily activities	2.81(2.4)				
Lost control of mood	3.06(2.5)				

*BDI-II* Beck Depression Inventory-II, *PSQI* Pittsburgh Sleep Quality Index

### Relationships among the variables

Table 3 shows that fatigue, sleep quality, and eGFR were significantly correlated with depression. The results indicated that the higher the levels of depression, the higher the probability of being fatigued, the poorer the sleep quality, and the lower the eGFR. Fatigue was significantly positively correlated with sleep quality and negatively correlated with eGFR. This finding showed that the more tired the participants, the lower their eGFR and the pooper their sleep quality. Furthermore, patients who reported better emotional and social support were more likely to have better sleep quality and lower degrees of fatigue and depression, although no significant correlation was observed between eGFR and emotional and social support.

**Table 3:** Correlations among ESS, FS, BDI-II, PSQI, and GFR (*n* = 242)

	FS	BDI-II	eGFR
PSQI	0.52**	0.61***	-0.50***
FS		0.48***	-0.39**
BDI-II			-0.64**

^**^p < 0.01; ***p < 0.001; *BDI-II* Beck Depression Inventory-II, *PSQI* Pittsburgh Sleep Quality Index, *FS* Fatigue scale

### Constructs of the intermediary model

**Fig. 2:**
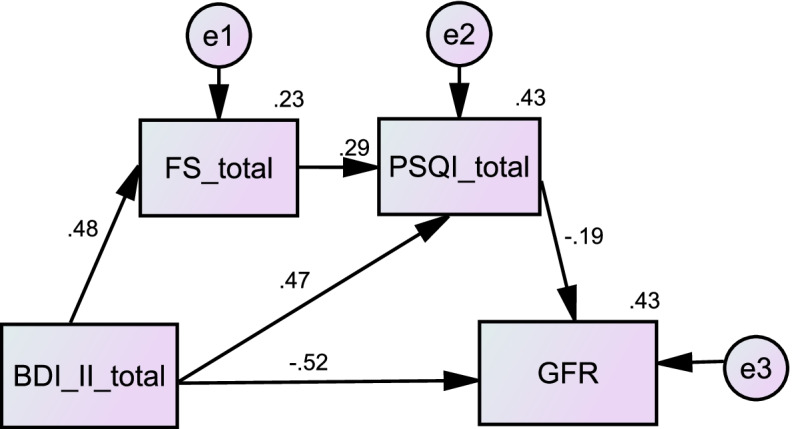
The SEM of predictors on eGFR in patients with CKD-ND. BDI-II: Beck Depression Inventory-II; PSQI: Pittsburgh Sleep Quality Index; eGFR: Estimate glomerular filtration rate; FS: Fatigue scale

A model was constructed to examine the direct and indirect effects of depression, fatigue, and sleep quality on eGFR (Fig. 2). Our initial conceptual model exhibited a lack of model fit. After some modifications, we removed the fatigue-mediated depression and eGFR pathways. The overall goodness-of-fit statistics showed that the proposed model fits the data well, with an RMSEA of 0.025, χ2 of 1.149, GFI of 0.998, and AGFI of 0.976. Figure 2 shows that sleep quality had a partial mediating effect on the relationship between depression and eGFR, and fatigue and sleep quality were mediators between depression and eGFR.

$$\mathrm{Chi}\_\mathrm{square}=1.149;\mathrm{ p}\_\mathrm{value}=0.284;\mathrm{ RMSEA}=0.025;\mathrm{ GFI}=0.998;\mathrm{ AGFI}=0.976$$

### Mediating effects of fatigue and sleep quality

The results of the analysis of the relationship between personal characteristics, depression, fatigue, and sleep quality of participants showed statistically significant differences in marital status, religious beliefs, living conditions, CKD stage, and ESS scores. Therefore, eGFR was the outcome variable, depression was the independent variable, fatigue and sleep quality were mediating variables, and the aforementioned variables were covariates entered in the model.

Table 4 shows that the indirect effect size was − 0.1738 (95% confidence interval [CI], − 0.2812 to − 0.0651), which was a statistically significant difference, indicating that the model has a mediating effect. The direct effect size and total effect size were − 0.6566 (95% CI, − 0.8191 to − 0.4942) and − 0.8304 (95% CI, − 0.9602 to − 0.7006), respectively, which were statistically significant differences, indicating that the overall model has a partial mediating effect. Three paths were further analyzed: (1) whether fatigue mediates depression and eGFR, (2) whether sleep quality mediates depression and eGFR, and (3) whether fatigue and sleep quality mediate depression and eGFR simultaneously. In Path 1, fatigue did not mediate depression and eGFR (95% CI, − 0.1292 to 0.0440). Path 2 showed that sleep quality was a significant mediating variable of depression and eGFR, and the indirect effect size was − 0.1058 (95% CI, − 0.2047 to − 0.0143). In Path 3, fatigue and sleep quality simultaneously mediated depression and eGFR, with an indirect effect size of − 0.0281 (95% CI, − 0.0587 to − 0.0039). Therefore, fatigue and sleep quality can indirectly affect the relationship between depression and eGFR.

**Table 4:** Analysis of the effect of multiple intermediaries

			95% CI		
	Effect	SE	lower	upper	*p*
**BDI-II → FS**	0.5874	0.0727	0.4442	0.7307	0.0000
**BDI-II + FS → PSQI**					
BDI-II	0.1711	0.0212	0.1293	0.2129	0.0000
FS	0.0733	0.0168	0.0442	0.1105	0.0000
**BDI-II + FS + PSQI → eGFR**					
BDI-II	-0.6566	0.0824	-0.8191	-0.4942	0.0000
FS	-0.0680	0.0604	-0.1869	0.0509	0.2609
PSQI	-0.6181	0.2242	-1.0598	-0.1764	0.0063
**Direct effect**	-0.6566	0.0824	-0.8191	-0.4942	0.0000
**Indirect effect**	-0.1738	0.0542	-0.2812	-0.0651	
**Total effect**	-0.8304	0.0659	-0.9602	-0.7006	0.0000
**Ind1**	-0.0400	0.0439	-0.1292	0.0440	
**Ind2**	-0.1058	0.0481	-0.2047	-0.0143	
**Ind3**	-0.0281	0.0137	-0.0578	-0.0039	

*BDI-II*: Beck Depression Inventory, *PSQI* Pittsburgh Sleep Quality Index, *eGFR* Glomerular filtration rate, *FS* Fatigue scale, Ind1: BDI-II → FS → eGFR; Ind2: BDI-II → PSQI → eGFR; Ind3: BDI-II → FS → PSQI → eGFR

## Discussion

The results of this study showed that 39% of patients with CKD-ND have depression problems, and 12% have severe depression. This result was similar to the 37% morbidity rate of depression in patients with CKD-ND found in a survey by Tsai et al. [10]. The rate of severe depression was slightly lower than that reported by Wang et al. [6], who found that approximately 23% of patients with CKD-ND have severe depression problems. This disparity can largely be explained by Wang et al.’s use of different measurement scales and different regions. Furthermore, most participants (44%) in this study had stage 3 CKD, and their BDI-II score was lower (9.9 ± 6.5). In our study, only patients with stage 5 CKD-ND (29%) had clinically significant depression (BDI-II: 28.1 > 17). Therefore, in this study, the percentage of severe depression in patients with CKD-ND was lower. The results of the analysis of the relationship between personal characteristics, depression, fatigue, and sleep quality showed statistically significant differences in marital status, religious beliefs, living conditions, CKD stage, and ESS scores. This result was similar to that of Chiang et al. [41], who found that CKD-ND patients who were depressed were also more likely to be single, living alone, and have no religious beliefs. Wang et al. also found that the incidence of depression was significantly associated with single, divorced, or widowed status [6]. Our inference was related to whether the patient had adequate social support. The study found that patients with ESRD who had more social support had a better physical quality of life and sleep quality and fewer depressive symptoms than those with less social support [29]. Social support is a promising strategy to decrease CKD-related depressive symptoms [42]. Patients who were married, had religious beliefs, and lived with spouse and children had more sources of social support. Therefore, it was inferred that their depression and fatigue scores will be lower, and their sleep quality will be better.

In this study, the depression scores of patients with stage 5 CKD-ND were significantly higher than those of patients with stage 3 and 4 CKD. This result was consistent with that reported by Tsai et al. and Tu et al., who found that patients with stage 5 CKD-ND had a significantly higher percentage of clinical depression than those with stage 3 CKD [10, 19]. Chiang et al. also found that patients with stage 4 or above CKD-ND were more than twice as likely to have depression [41]. One of the possible reasons for the higher proportion of clinically significant depression in patients with stage 5 CKD-ND originates from the expectation of dialysis. The fear of entering dialysis treatment and worries about changing health status further aggravate depression. HCPs should pay attention to this issue because the existence of depression may affect the disease management behavior of patients with stage 5 CKD-ND and patients may show poor adherence behaviors and respond poorly to treatment [6]. Our study found that although the degree of fatigue of patients with all stages of CKD-ND was not high, patients with stage 5 CKD-ND still had a higher fatigue score than others. This result is similar to the results reported by Jhamb et al., who found that patients with advanced non-dialysis-dependent CKD experienced profound fatigue [21]. This was in line with the expectation that patients with early CKD-ND were more functional and less fatigued than those with advanced CKD-ND. It may be that kidney-disease-related factors such as anemia and low albumin contribute to fatigue among patients with advanced CKD-ND more than those with early CKD-ND [21, 22]. Approximately 91% of patients in this study had poor sleep quality, which was much higher than that (36.2%) reported by Tu et al. [19]. Our study found that patients with all stages of CKD-ND had poor sleep quality, especially those with stage 5 CKD-ND. According to relevant studies, the poor sleep quality of patients with CKD-ND is related to kidney-disease-related factors such as pruritus and restless legs syndrome, which were commonly seen in patients with stage 5 CKD-ND [43, 44]. Thus, their sleep quality was generally poorer than that of others stages.

This study found that depression was positively correlated with sleep quality (*r* = 0.61, *p* < 0.001) and fatigue (*r* = 0.48, *p* < 0.001), indicating that the more severe the depression in patients with CKD-ND, the poorer the sleep quality and the higher the level of fatigue. This result was similar to that of Chiang et al. [41], who found that depression was significantly associated with sleep disturbances. Similarly, Jhamb et al. [21] indicated that depression was significantly positively associated with the fatigue of patients with CKD-ND. Sleep disorders and depression are closely related and complex. Sleep disturbance is one of the key symptoms of depression [20], and depression may result in sleep disturbance [12, 19]; thus, they may have an influence on each other [16]. Poor subjective sleep quality was significantly associated with high levels of fatigue, and fatigue was also positively associated with depressive symptoms [21]. However, whether a causal mechanism exists between the three is uncertain. Elucidating this intricate relationship and the direction of effects may help in the development of more effective mechanisms to address depression in patients with CKD-ND.

Depression (*r* =  − 0.64, *p* < 0.01), fatigue (*r* =  − 0.39, *p* < 0.01), and sleep quality (*r* =  − 0.50, *p* < 0.001) were negatively correlated with eGFR, showing that CKD-ND patients with higher levels of depression, fatigue, and poor sleep quality also had poorer renal function. These results agreed with previous research results [6, 7, 13, 45–47]. Fatigue was independently associated with ESRD progression, death, or hospital admission in patients with CKD-ND [22, 23]. Moderate or severe depression increases the risk of eGFR reduction by approximately 1.7 times [45]. Shorter sleep duration and poorer sleep quality may promote CKD progression and adversely affect renal function [7, 47]. A 4-year prospective generation study by Yamamoto et al. highlighted that short sleep time and poor sleep quality are significantly associated with a higher incidence of ESRD [46]. The results of this study are similar to those of Ogna et al., who found that the lower the eGFR, the lower the quality of sleep [13]. Furthermore, although other studies have found no significant correlation between eGFR and sleep quality in patients with CKD-ND [48], patients with poor sleep quality are more likely to experience proteinuria [9, 15, 37], thereby worsening their renal function.

The results of this study showed that depression was closely correlated with eGFR, and the relationship between depression and eGFR was mediated by fatigue and sleep quality. First, sleep quality had a partial mediating effect on the relationship between depression and eGFR. Current studies have established the relationship between sleep quality and several physiological systems that affect renal function [7, 47]. The circadian timing system is critically implicated in maintaining physiological functions related to the kidney, including blood pressure and eGFR [37, 49]. Evidence shows that poor sleep quality may lead to sympathetic nerve stimulation, thereby adversely affecting renal hemodynamics and blood pressure [50], reducing plasma renin activity, and leading to an imbalance of aldosterone regulation, which can lead to kidney damage [7, 15]. Moreover, depression may interfere with sleep quality, and sleep disturbance is one of the key symptoms of depression [51]. Therefore, it is inferred that depressive symptoms will affect sleep status, which indirectly affects renal function. Conversely, pathological mechanisms shared by the kidney and brain tissue damage, such as the renin–angiotensin system, may contribute to cerebrorenal interactions and exacerbate depression in patients with CKD [42]. Second, although fatigue was statistically significantly associated with depression and eGFR, it had no mediating effect on the relationship between depression and eGFR. The reason for this may be that most of the participants had stage 3 CKD-ND in this study and had no significant kidney-disease-related symptoms, so the fatigue level was lower. Third, after sleep quality was added between depression, fatigue, and eGFR, fatigue and sleep quality showed a partial mediating effect on the relationship between depression and eGFR. Fatigue and sleep disturbances are among the criteria that define depression [20, 23, 28]. Although their relationship is closely related and complex, our findings were supported by a longitudinal study by Rodgers et al. who indicated that the impact of depression on health-related quality of life (HRQoL) would be mediated by fatigue [52]. Disappointed and depressed about illness, can worsen patients' emotional state, make them feel fatigued, decrease vitality and even affect their sleep quality [23]. Once when illness or treatment-related fatigue interferes with patients’ occupational and interpersonal functions, they may feel disappointed and depressed, which may worsen their emotional state, which in turn impair the HRQoL of patients with CKD-ND [23, 43]. A previous study had proposed a biopsychosocial model of fatigue in patients with ESRD. In this model, fatigue results from biochemical imbalances or stress and worry; depression and anxiety cause maladaptive behavior patterns (e.g., sleep problems) through negative beliefs; and maladaptive behavior patterns will cause negative beliefs, leading to depression and anxiety, which subsequently aggravate the patient’s biochemical imbalance and pressure and create a vicious cycle of maintenance [53]. The aforementioned explanation may support the study findings that fatigue and sleep quality partially mediate depression and eGFR. In addition to psychosocial factors, biochemical and hematological factors, such as chronic inflammation. Inflammatory cytokines have been suggested to have an important role in the onset of fatigue in renal patients, it may result in fatigue in patients with CKD-ND, indirectly influence sleep [54]. Although the causal relationship between depression, fatigue, and sleep disturbance is difficult to define. In this study, we tried to use a mediation model to explain their relationship. However, more empirical research results are needed to confirm the causal relationship in the future.

### Limitations

Our study had some limitations that must be mentioned. First, because convenience sampling was adopted and geographical limitations might affect the external validity of the research results, caution should be used when extending our results to a larger population. Second, the subjectivity of the self-report questionnaire is noteworthy. Third, our study lacked objective sleep data or the impact of OSA. Tu et al. indicated that objective sleep quality may be more heavily influenced by renal-function-related factors, and the composite severity of subjective sleep disturbance is subjective to renal-function-related and psychological factors [19]. Because our variables are mainly psychological factors, we believe that subjective sleep quality may be more in line with our research topic. Finally, the cross-sectional nature of this study was a limitation, which clearly does not allow the establishment of temporal precedence of the mediators. Although we collected the patients’ eGFR for the next month, the time effect of depression, fatigue, and sleep quality was still unclear. There is a very strong association between sleep disturbance and major depressive disorder. Sleep disturbance is one of the main symptoms of the disease, and it is very likely that both exist at the same time [20]. In addition, one of the nine diagnostic domains of major depressive disorder is the presence of fatigue for at least 2 weeks [23]. For these reasons, it is reasonable to assume that depression, sleep disturbance, and fatigue may co-occur and be difficult to distinguish in patients with CKD-ND. Therefore, we thought that it might be feasible to collect depression, fatigue, and sleep quality status simultaneously. Future research using longitudinal designs is clearly warranted to draw causal conclusions.

## Conclusions

How to improve disease management in patients with CKD-ND to delay the start of dialysis is clinically challenging. This study was mainly designed to explore whether fatigue and sleep quality were mediating variables between depression and renal function. The results showed that fatigue and sleep quality exhibited a partial mediating effect on the relationship between depression and eGFR. This result provided preliminary evidence to support the hypothesis of a mediating variable between depression and renal function. Therefore, the predictors of renal function decline, such as depression, fatigue, and sleep quality, that affect patients with CKD-ND should be assessed and improved. Considering the mediating effect of fatigue and sleep quality between depression and renal function, we suggest that HCPs should regularly screen for depressive symptoms in patients with CKD-ND. When assessing patients’ depressive state, sleep quality and fatigue should also be included. It should be helpful to seek psychiatric evaluation and treatment when managing depressive symptoms of patients with CKD-ND. However, research evidence indicates that depression, fatigue, and sleep disturbance in patients with CKD-ND are multifactorial and complex [21, 22]. Whether psychological and physiological interventions for depression, sleep, and fatigue could effectively improve the renal function of patients with CKD-ND is worthy of further investigation.

## Data Availability

The datasets used and analyzed during the present study are available from the corresponding author on reasonable request.

## Abbreviations

AGFI: Average GFI
BDI-II: Beck Depression Inventory-II
CI: Confidence interval
CKD: Chronic kidney disease
CKD-EPI: CKD Epidemiology Collaboration
CKD-ND: Nondialysis chronic kidney disease
ESRD: End-stage renal disease
ESS: Emotional and Social Support Scale
FS: Fatigue Scale
eGFR: Glomerular filtration rate
GFI: Goodness-of-fit index
HCPs: Help healthcare providers
HRQoL: Health-related quality of life
MDRD: Modification of Diet in Renal Disease
OSA: Obstructive sleep apnea
PSQI: Pittsburgh Sleep Quality Index
REM: Rapid eye movement
RMSEA: Root mean square error of ap-proximation
